# PEpiD: A Prostate Epigenetic Database in Mammals

**DOI:** 10.1371/journal.pone.0064289

**Published:** 2013-05-16

**Authors:** Jiejun Shi, Jian Hu, Qing Zhou, Yanhua Du, Cizhong Jiang

**Affiliations:** Shanghai Key Laboratory of Signaling and Disease Research, Department of Bioinformatics, Shanghai Tenth People's Hospital, The School of Life Sciences and Technology, Tongji University, Shanghai, China; Roswell Park Cancer Institute, United States of America

## Abstract

Epigenetic mechanisms play key roles in initiation and progression of prostate cancer by changing gene expression. The Prostate Epigenetic Database (PEpiD: http://wukong.tongji.edu.cn/pepid) archives the three extensively characterized epigenetic mechanisms DNA methylation, histone modification, and microRNA implicated in prostate cancer of human, mouse, and rat. PEpiD uses a distinct color scheme to present the three types of epigenetic data and provides a user-friendly interface for flexible query. The retrieved information includes Refseq ID, gene symbol, gene alias, genomic loci of epigenetic changes, tissue source, experimental method, and supportive references. The change of histone modification (hyper or hypo) and the corresponding gene expression change (up or down) are also indicated. A graphic view of DNA methylation with exon-intron structure and predicted CpG islands is provided as well. Moreover, the prostate-related ENCODE tracks (DNA methylation, histone modifications, chromatin remodelers), and other key transcription factors with reported roles in prostate are displayed in the browser as well. The reversibility of epigenetic aberrations has made them potential markers for diagnosis and prognosis, and targets for treatment of cancers. This curated information will improve our understanding of epigenetic mechanisms of gene regulation in prostate cancer, and serve as an important resource for epigenetic research in prostate cancer.

## Introduction

Prostate cancer (PC) is the most common form of noncutaneous cancer and a prominent cause of cancer death in men. The process of PC involves a number of genetic variations and gene expression changes such as mutation, polymorphism, gene amplification, and so on. However, in addition to genetic alteration, epigenetic aberration also plays key roles in progression of PC. The major epigenetic factors include DNA methylation, histone modification, and non-coding RNAs, especially microRNA [1]. DNA methylation modifies DNA by adding a methyl group to the 5′-carbon of cytosine in CpG dinucleotide. It often occurs in CpG islands in the promoter regions of genes [2]. Abnormal DNA methylation (hypo- or hypermethylation) can cause chromatin structure change that in turn may lead to transcriptional gene silencing, predisposition to mutation and allelic deletions. Both type of the abnormal DNA methylation have been implicated in tumor development [3]. The androgen receptor (AR) is critical for the normal development and function of prostate. The aberrant methylation in AR promoter CpG islands was observed in prostate cancer cells [4].

Nucleosome, a histone octamer wrapped by about 147bp DNA, is the fundamental unit of chromatin. The N-terminal tails of histones are subject to different covalent modifications. The modifications affect chromatin remodeling and may activate or inactivate gene transcription depending on the location and type of the modification. A previous study reported the reduced histone acetylation or H3K4me2 and increased H3K9me2 in the hypermethylated promoter of RASSF1A gene. Moreover, the pattern of histone modifications helped to maintain RASSF1A gene silencing with its promoter aberrant DNA methylation in prostate cancer [5].

MicroRNAs, a class of∼22nt non-coding RNAs, control gene expression post-transcriptionally by degrading or repressing translation of target mRNAs through perfect or imperfect sequence match. This regulation of gene expression by microRNAs has a large impact on the components of transcriptome and proteome of eukaryotic species. The aberrant microRNA expression has been associated with cancers. MicroRNA expression profiling of 6 prostate cancer cell lines identified 51 individual microRNAs whose expression was significant different between benign tumors and carcinoma tumors. Clustering analysis of these microRNA expression was able to classify the prostate carcinoma tumors to androgen dependence or independence [6].

Studies on epigenetic changes in PC have accumulated vast amount of useful data that could shed light on the development of PC and include potential epigenetic markers as diagnosis and prognosis for PC. However, none of the existing databases provide epigenetic changes involved in prostate cancer. Therefore, in this study, we retrieved the useful epigenetic data through literature mining, integrated related knowledge, provided cross-references to other databases, and then built a web-based prostate epigenetic database (PEpiD). PEpiD will serve as a useful resource for research in PC.

## Methods

PEpiD is a relational database with a MySQL data layer. The interaction between data and application comprises Python and PHP modules. The presentation of the data with graphical user interface was developed by DHTML and JavaScript.

## Results and Discussion

### Overview of the Database

PEpiD stores three types of epigenetic data (DNA methylation, histone modification, microRNA) which previous studies indicated as involved in prostate cancer of human, mouse, and rat. The data were retrieved from our exhaustive literature curation. The epigenetic status or changes collected in PEpiD were experimentally verified as being involved in PC. The external links to the supportive literatures are provided. For the integrity of knowledge, cross-references to other related databases such as PubMed, Entrez Gene, and miRBase [7] were established in PEpiD.

In order to efficiently access the data, PEpiD has a user-friendly interface and provide users with multiple search methods to retrieve the information of interest. PEpiD combines both text-compiled presentation and graphic view of query results for clarity. An upload function is available in PEpiD for users to submit the newly identified epigenetic changes in PC. This paves a way for PEpiD to grow in content by addition of the newly findings in time.

### Data Access and Utility

PEpiD provides a concise query page for users to access the database. Based on the information which users likely to know about the epigenetic changes in PC, users have several query methods to search PEpiD including species, Refseq ID, gene symbol, genomic location, specimen, and assay generating the epigenetic data. The query methods are organized into three blocks. Block I consists of species, Refseq ID, gene symbol, genomic location. Block II and III contain phenotype and assay, respectively. The query methods in the three blocks can be combined with Boolean operation AND or OR. It should be noted that Refseq ID, gene symbol, and genomic location in block I are exclusive. Namely, when a Refseq ID is given, gene symbol and genomic location are automatically unavailable, *vice versa*. Such query method settings allow both loose and strict search.

**Figure 1:**
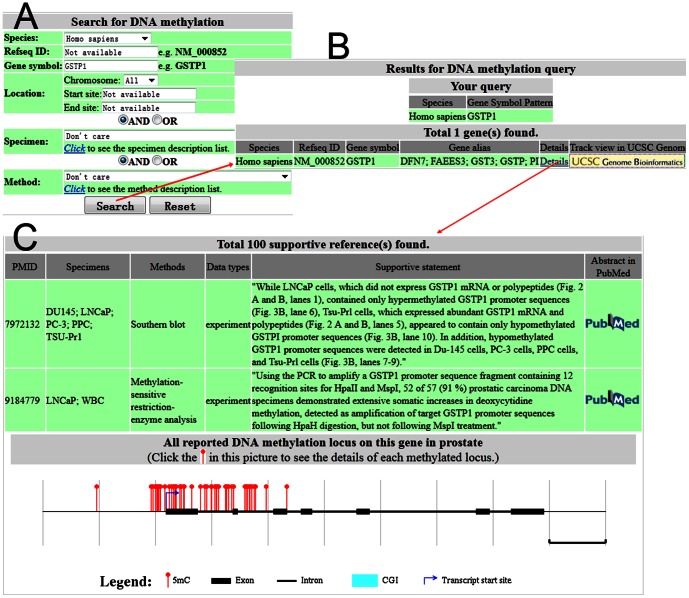
Screen shots showing DNA methylation query by Gene symbol GSTP1 in human. A, the search form page in which the exclusion relationship between Refseq ID, Gene symbol, and Location is indicated. B, the query result page embedded with the link to track view in UCSC genome browser. C, the details page showing supportive references and DNA methylation loci in a graph.

Here, we use a query in DNA methylation in PC as an example to present the query and its output. The loosest search is to retrieve all DNA methylation loci by leaving all parameters blank. When specifying start and end sites on a chromosome, one can obtain all DNA methylation loci in the specified genomic region. One can retrieve the DNA methylation loci occurred in the specified tissue or cell line, for example, DNA methylation loci observed in the cell line LNCaP by choosing LNCaP in the option specimen. More filtering can be added this way for accurate query, for example, setting MeDIP in the experimental assay to retain DNA methylation loci generated by MeDIP. At most time, one is interested in the DNA methylation change in a certain gene implicated in PC. For the purpose, users can input the Refseq ID or gene symbol of interest for query (Fig. 1A). Note that gene symbol will also look up the gene alias table in our database to catch all possible matched genes. Addition to this, both Refseq ID and gene symbol allow fuzzy query for flexibility. On the query result page (Fig. 1B), the top table is the summary of the query settings. The bottom table lists the genes satisfying the query settings. Users can click the “Details” to get the complete information for the hit gene. The “Details”page has three parts (Fig. 1C). The first part displays the basic information for the hit gene such as its alias, genomic location, brief description, crosslink to Entrez Gene database, and link to track view in UCSC genome browser (not shown). The second part gives supportive references together with the specimen, experimental method, data type in the literature and its PubMed link. Moreover, the essential supportive statement is retrieved from the literature and given in the second part. The last part is a graphic view of the gene structure with displaying the exons, introns, transcription start site (TSS), CpG islands (CGI) if any, and DNA methylation loci. The genome-wide CpG islands were predicted by CpGcluster [8]. The click on the DNA methylation locus can lead to the genomic sequences with the methylated C marked in red.

**Figure 2:**
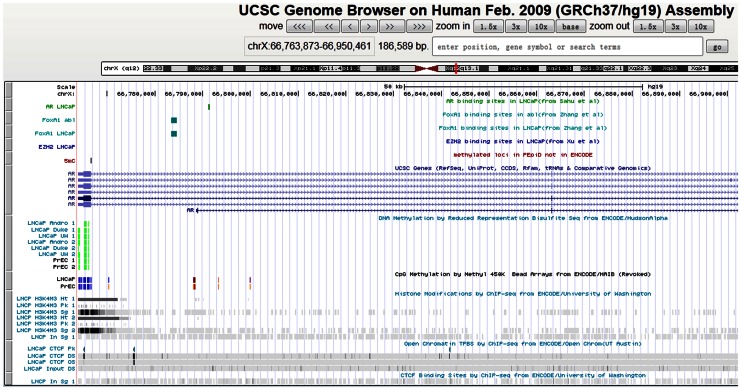
Track view of prostate specific epigenetic marks in gene AR. The top block in the track view shows our curated regulators AR, FoxA1, EZH2, and DNA methylation loci (not from ENCODE project). Then it follows UCSC gene track, DNA methylation, histone modifications, and CTCF binding sites from ENCODE project, respectively.

In order to obtain the complete information pertaining to the epigenetic regulation in prostate cancer, we retrieved the binding sites of important regulators with reported roles in prostate cancer, such as transcription factors AR [9] and FoxA1 [10], and the catalytic subunit of Polycomb repressive complex 2 (EZH2) [11], and prostate specific ENCODE tracks from UCSC genome browser. Then, we organized our curated data, prostate specific ENCODE tracks, and the transcription factors in tracks and displayed them in UCSC genome browser. In this “track view” page (Fig. 2), our curated data is shown at the top followed by UCSC gene track relevant ENCODE tracks (DNA methylation, histone modifications, and CTCF sites). This page provides the relative complete view of the epigenetic marks in the specified gene or region. Such graphic views will largely facilitate scientists to examine the state of epigenetic modifications in the gene or region of interest.

Various histone modifications occur at different positions of histone tails. Therefore, an additional query method “modification type” was designed for histone modification. When setting “modification type” to H3K4me3, users can find all H3K4me3 in PC and associated genes. Moreover, the histone modification change (hyper or hypo) is indicated if such information is available. The resultant gene expression regulation (up or down) is also shown in the “Details” page. This information gives users clear and full picture of the histone modification change and the impact on gene regulation.

A microRNA often regulates more than one target genes. A gene is also regulated by multiple microRNAs. According to the regulation relationship between microRNAs and target genes, PEpiD allows users to input a target gene and find its upstream regulator microRNA(s) involved in PC. Because miRBase [7] collects relative complete information for microRNAs, an external link to miRBase for hit microRNA is established in PEpiD. Thus, users can have the sequences of hairpin and mature microRNAs, and the stem-loop hairpin structure.

In order to catch the new findings in epigenetic studies in PC and to expand PEpiD by adding the new findings in time, we provide a “submit” window for researchers to submit their new findings to PEpiD. We will regularly manually curate the submissions and add the approved data to PEpiD. The “submit” function provides a way for peer scientists in PC to efficiently share their new findings in PEpiD.

### Comparison to Other Related Databases

To date, there are three databases storing the useful data pertaining to PC from published work. The prostate expression databases (PEDB (human) and mPEDB (mouse)) archive prostate gene expression information from 44 and 16 prostate cDNA libraries, respectively. The gene expression data is comprised of expressed sequence tags, and microarray data. PEDB provides detailed library information such as tissue source, sequence diversity, and more [12]. The prostate gene database (PGDB) is a repository of genes and genomic loci related to the human prostate and prostatic diseases. PGDB contains not only the genes specifically expressed in prostate but also the genes implicated in various molecular and genetic events of the prostate such as mutation, polymorphism, over-expression, and so on. Currently, PGDB has only 165 unique entries [13]. Another database, dragon database of genes associated with prostate cancer (DDPC) stores genes that have been experimentally verified as involved in PC. For each gene, DDPC integrated all kinds of related information together including molecular interactions, pathways, gene ontologies, predicted transcription factor binding sites on the promoter, transcription factors potentially binding to these sites, and drug-related information. Basically, DDPC is an integrated knowledgebase of genes implicated in PC [14].

Studies have shown that epigenetic gene regulation has important roles in tumor initiation and progression. However, none of the above databases provides epigenetic changes involved in prostate cancer. We also checked other epigenetic databases. PubMeth is a cancer methylation database and has only 80 PC-related genes [15]. MethDB is another DNA methylation database contains only two genes (GSTP1 and RASSF1) in PC and seven references [16]. HHMD is a human histone modification database collects 394 histone modifications in 330 genes [17]. miR2Disease is a curated database on microRNA in human disease and includes 96 microRNAs involved in PC [18]. To date, research in PC has let to vast amount of epigenetic data of PC. Therefore, a curated database on epigenetic changes in PC will provide insights into the epigenetic mechanisms of gene regulation in PC. PEpiD complements the currently available PC-related databases (PEDB, mPEDB, PGDB, DDPC), the entries of epigenetic changes in PC in PEpiD largely exceed the above databases (Table 1), and will serve an important resource for study in PC at an epigenetic level.

**Table 1:** Number of epigenetic records and genes in PEpiD, with gene numbers in parentheses.

Species	DNA methylation	Histone modification	microRNA
Human	458066 (18920)	35093 (13959)	718 (86)
Mouse	69 (44)	8 (8)	105 (4)
Rat	12 (12)	2 (2)	0 (0)

### Current Status and Future Developments

The data in PEpiD came from our exhaustive literature mining and curation. Briefly, we designed extensive and typical query configurations to retrieve all possible literatures related to epigenetic study in PC. For example, we used the query (“prostate”[All Fields]) AND ((“dna”[All Fields] AND “methylation”[All Fields]) OR “dna methylation”[All Fields]) to obtain all literatures studying on DNA methylation in PC. In the end, we collected and manually read more than 1400 MEDLINE records, retrieved and curated the useful information, then added to PEpiD. In summary, the current release of PEpiD contains more than 800,000 epigenetic records and 18,000 genes implicated in PC that are supported by 658 MEDLINE records (Table 1). There are total of 86 target genes regulated by 718 microRNAs indicated to be involved in human PC. Only 96 microRNAs were reported to have at least one target gene. With more target genes to be validated in the future, more microRNAs will be confirmed to have more than one target genes.

In order to integrate the related epigenetic data newly identified in PC-related studies in time, we will update PEpiD on content and functionality annually. Ongoing work includes addition of the newly identified epigenetic data in PC to PEpiD and update of the entries present in PEpiD. Studies have shown the crosstalks between the different epigenetic factors. Therefore, the association of DNA methylation, histone modification, and microRNA implicated in PC will be a future development for PEpiD. This future will improve the integrity of knowledge in PEpiD and provide users with more useful and advanced information about epigenetic mechanisms in PC. Although there are other databases regarding PC, PEpiD is the only one archiving the epigenetic changes and the associated genes in PC. PEpiD will facilitate scientists to investigate epigenetic mechanisms underlying progression of PC, to identify potential epigenetic markers for diagnosis, prognosis of PC and even drug target.

## Conclusions

PEpiD is a PC-specific epigenetic database consisting of DNA methylation, histone modification, and microRNA experimentally verified to be involved in PC. The essential information associated with the epigenetic changes is integrated in PEpiD such as tissue source, experimental method, CpG islands, gene structure, corresponding sequences, and supportive references. Moreover, the impact of epigenetic changes on gene expression (up- or down-regulated) is also documented in PEpiD. For completeness of related information, cross-references to external well-known databases are established in PEpiD. Especially, the prostate related ENCODE tracks and transcription factor binding sites are displayed together with our curated data in UCSC genome browser. The comprehensive knowledge of epigenetic changes implicated in PC in PEpiD can facilitate scientists to search epigenetic changes individually by type or feature, and further examine the crosstalks between epigenetic factors. Thus, this database will be an important resource for research in the field of PC, and improve our understanding of epigenetic mechanisms in PC.
