# Correction: PEpiD: A Prostate Epigenetic Database in Mammals

**DOI:** 10.1371/annotation/1236c3ed-1497-4917-a083-2f5be4fab835

**Published:** 2013-10-23

**Authors:** Jiejun Shi, Jian Hu, Qing Zhou, Yanhua Du, Cizhong Jiang

Affiliation number 1 for all authors is incorrect. The correct affiliation is: Shanghai Tenth People's Hospital, Department of Bioinformatics, Shanghai Key Laboratory of Signaling and Disease Research, the School of Life Sciences and Technology, Tongji University, Shanghai, 200092, China. 

